# Chromoselective Photocatalysis Enables Stereocomplementary Biocatalytic Pathways**

**DOI:** 10.1002/ange.202100164

**Published:** 2021-02-26

**Authors:** Luca Schmermund, Susanne Reischauer, Sarah Bierbaumer, Christoph K. Winkler, Alba Diaz‐Rodriguez, Lee J. Edwards, Selin Kara, Tamara Mielke, Jared Cartwright, Gideon Grogan, Bartholomäus Pieber, Wolfgang Kroutil

**Affiliations:** ^1^ Institute of Chemistry Department of Organic and Bioorganic Chemistry University of Graz, NAWI Graz, BioTechMed Graz Heinrichstrasse 28 8010 Graz Austria; ^2^ Department of Biomolecular Systems Max Planck Institute of Colloids and Interfaces Am Mühlenberg1 14476 Potsdam Germany; ^3^ Chemical Development, Medicinal Science and Technology, Pharma R&D GlaxoSmithKline Medicines Research Centre Gunnels Wood Road Stevenage SG1 2NY UK; ^4^ Department of Engineering, Biological and Chemical Engineering Biocatalysis and Bioprocessing Group Aarhus University Gustav Wieds Vej 10 8000 Aarhus Denmark; ^5^ Department of Chemistry University of York Heslington York YO10 5DD UK; ^6^ Field of Excellence BioHealth- University of Graz 8010 Graz Austria

**Keywords:** carbon nitrides, C−H activation, chromoselectivity, photobiocatalysis, unspecific peroxygenases

## Abstract

Controlling the selectivity of a chemical reaction with external stimuli is common in thermal processes, but rare in visible‐light photocatalysis. Here we show that the redox potential of a carbon nitride photocatalyst (CN‐OA‐m) can be tuned by changing the irradiation wavelength to generate electron holes with different oxidation potentials. This tuning was the key to realizing photo‐chemo‐enzymatic cascades that give either the (*S*)‐ or the (*R*)‐enantiomer of phenylethanol. In combination with an unspecific peroxygenase from *Agrocybe aegerita*, green light irradiation of CN‐OA‐m led to the enantioselective hydroxylation of ethylbenzene to (*R*)‐1‐phenylethanol (99 % *ee*). In contrast, blue light irradiation triggered the photocatalytic oxidation of ethylbenzene to acetophenone, which in turn was enantioselectively reduced with an alcohol dehydrogenase from *Rhodococcus ruber* to form (*S*)‐1‐phenylethanol (93 % *ee*).

## Graphical abstract

Green or blue? The wavelength controls the outcome. Chromoselective activation of a carbon nitride generates electron holes with different oxidation potentials that are used for selective oxidation processes. By coupling the photocatalytic reactions with biocatalysis the (*S*)‐ or the (*R*)‐enantiomer of 1‐phenylethanol was synthesized stereoselectively.

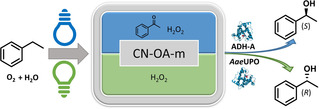

Many parameters influence the selectivity of a chemical reaction.^[1]^ For instance, catalytic reactions can be controlled by varying the catalyst/coordinated ligands, directing groups^[2]^ or by tuning external parameters (Scheme 1 A).[^1a^, ^3^] The selectivity of photochemical reactions varies with different wavelengths,^[4]^ but examples that use this for visible‐light photocatalysis are rare.^[5]^

**Scheme 1:**
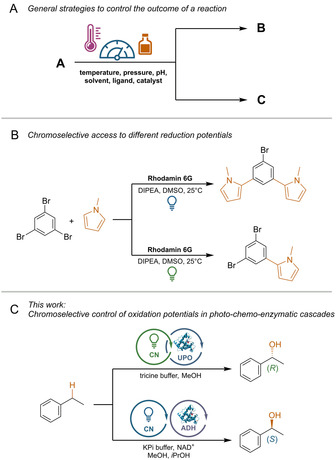
A) General approaches to control of the outcome of a chemical reaction. B) Chromoselective control in photocatalytic C−H‐arylations.^[5a]^ C) This study: Chromoselective control of the stereochemical outcome of photo‐chemo‐enzymatic reactions.

In one example, selective control between either a one‐ or two‐fold substitution of 1,3,5‐tribromobenzene with *N*‐methylpyrrole using Rhodamin 6G (Rh‐6G) as photocatalyst was demonstrated (Scheme 1 B).^[5a]^ This selectivity switch is explained by the chromoselective generation of two photocatalytic species that differ in their reduction potential. Green light irradiation results in a common photoredox cycle and the expected mono‐substituted product. In the case of blue light, the Rh‐6G radical anion, which is formed after quenching of Rh‐6G* with a sacrificial electron donor, can absorb a second photon, resulting in the highly reducing Rh‐6G^.−^* species that enables the formation of the di‐substituted product.^[5a]^

Here we show that electron holes with different oxidation potentials can be generated by using a heterogeneous carbon nitride (CN) catalyst by changing the incident photon energy. The combination of this strategy with two enantioselective biocatalysts^[6]^ allowed us to selectively produce the (*S*)‐ or (*R*)‐enantiomer of a chiral alcohol in photo‐chemo‐enzymatic reaction sequences (Scheme 1 C).

We recently realized that the choice of the wavelength is crucial for high selectivities in metallaphotocatalytic cross couplings using a heterogeneous carbon nitride material, which is made from urea and oxamide in molten salt (CN‐OA‐m).[^5b^, ^5c^, ^7^] While this can be rationalized by a purely kinetic effect, there is also evidence that a wavelength‐controlled generation of excited species with different oxidation potentials might be responsible for this phenomenon. CN‐OA‐m has a strong absorption up to ≈460 nm and a comparably weaker absorption band up to ≈700 nm, which were ascribed as the π–π* and n–π* electron transitions, respectively (Figure 1 A).^[8]^ The selective induction of the n–π* electron transition using long wavelengths (525 nm) should result in electron holes with a lower oxidation potential compared to irradiation using blue light (440 nm). The choice of the wavelength should not affect the reduction potential of the electron that is promoted into the valence band. Although such a behavior was previously suggested,^[8]^ there is, to the best of our knowledge, no report that applies this concept for controlling the selectivity of chemical reactions.

**Figure 1:**
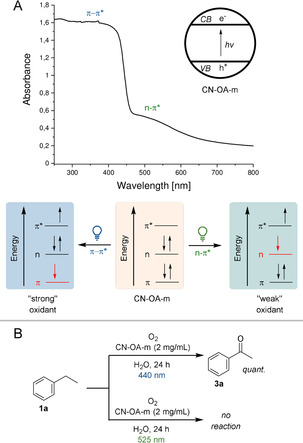
Chromoselective generation of excited CN‐OA‐m species with different oxidation potentials. A) Switching between π–π* and n–π* electron transitions using different wavelengths. B) The oxidation of ethylbenzene **1 a** to acetophenone **3 a** is only possible using blue light.

We hypothesized that such a strategy would allow us to induce a photocatalytic reaction of a substrate with green light selectively in the presence of a second compound that is only photo‐oxidized when shorter wavelengths are used. The photocatalytic aerobic oxidation of benzylic sp^3^ C−H bonds, which is feasible with other members of the carbon nitride family and blue light irradiation,^[9]^ served as a model reaction for our initial studies. In a series of experiments, we were indeed able to show that only blue light results in the desired carbonyl products and no reaction occurs at longer wavelengths (Figure 1 B).

Carbon nitrides are used to catalyse the formation of O_2_ and H_2_ via water oxidation^[10]^ and the production of hydrogen peroxide from oxygen and alcohols, which requires the reduction of O_2_.^[11]^ Hydrogen peroxide can then be used as stoichiometric oxidant in the enantioselective hydroxylation of ethylbenzene derivatives catalysed by the unspecific peroxygenase (UPO)^[12]^ from *A. aegerita* ^[13]^ (*Aae*UPO) acting as chiral catalyst.^[14]^

We hypothesized that a chromoselective activation of CN‐OA‐m with green light enables the selective formation of H_2_O_2_ in the presence of ethylbenzene (**1**) and the *Aae*UPO, which in turn catalyses the asymmetric hydroxylation of **1** (Figure 2). Performing the reaction in tricine buffer using 528 nm LEDs indeed resulted in a high selectivity towards (*R*)‐1‐phenylethanol formation [(*R*)‐**2 a,** up to 3.8 mM, 98 % *ee*] with low amounts (3 %) of acetophenone (**3 a**). When the same reaction was carried out using shorter wavelengths, **3 a** became the main product, thus supporting our hypothesis. Ketone (**3 a**) formation was also the preferred reaction in the presence of blue light in phosphate buffer. It is worth to note, that the type of buffer had a significant influence on the outcome on the reaction, whereby the molecular reason needs to be clarified.

**Figure 2:**
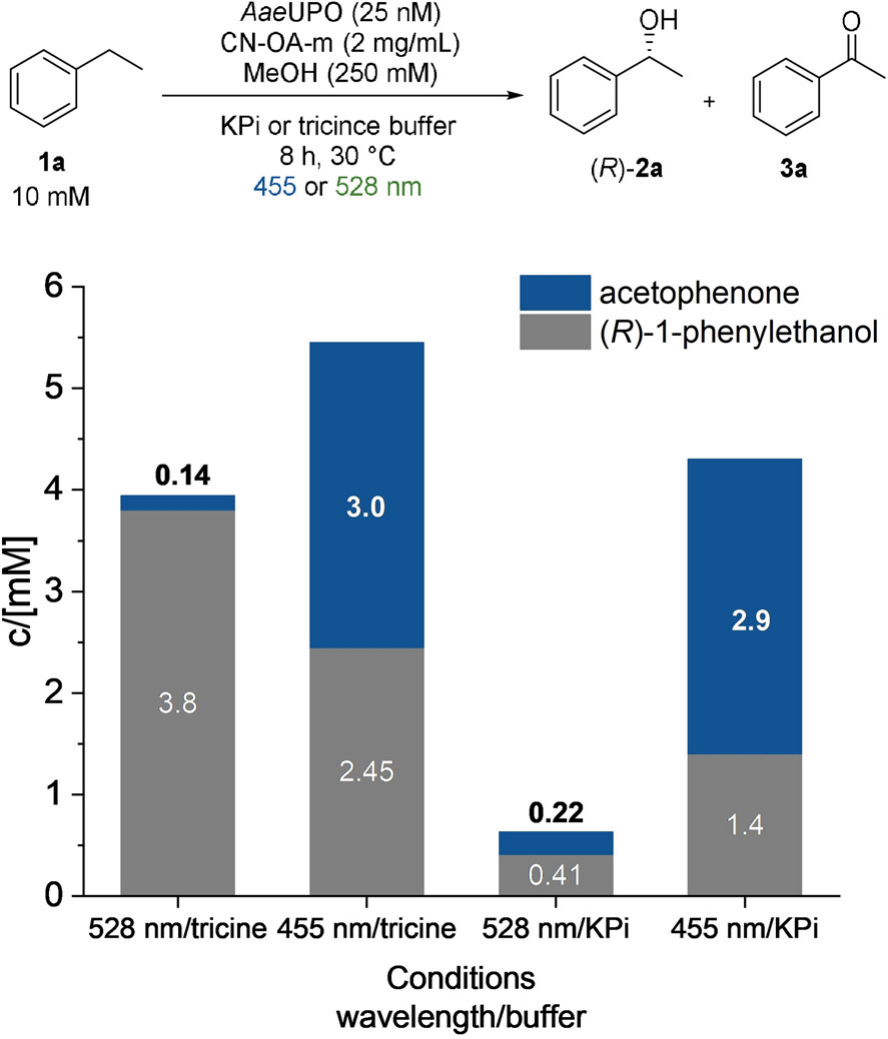
Influence of different wavelengths and buffers on the photo‐chemo‐enzymatic hydroxylation of ethylbenzene; reaction conditions: *Aae*UPO (25 nM), ethylbenzene (10 mM), CN‐OA‐m (2 mg mL^−1^), MeOH (250 mM), KPi (100 mM, pH 7.5) or tricine (100 mM, pH 7.5), 455 nm (1440 μmol photons m^−2^ s^−1^) or 528 nm (1330 μmol photons m^−2^ s^−1^), 30 °C, 8 h.

It was previously shown that UPOs are deactivated in the presence of blue light, a photocatalyst and O_2_ due to the generation of reactive oxygen species (ROS) that harm the enzyme.^[15]^ Consequently, one might expect that green light might be less harmful to the UPO and lead to higher conversions in comparison to blue light. To investigate this aspect, UPO and CN‐OA‐m were incubated for one hour in the presence of oxygen and green or blue light, before **1 a** was added (Figure S48). The mixture incubated at longer wavelengths indeed led to a higher conversion for the asymmetric hydroxylation after addition of **1 a**.

The milder conditions subsequently allowed an extension of the substrate scope for *Aae*UPO (Scheme 2). Nine additional substrates were converted with high stereoselectivity (>98 % *ee*) to the corresponding alcohols with concentrations of 1.0–6.0 mM. None of these ethylbenzene derivatives has been transformed with *Aae*UPO using an in situ H_2_O_2_ generation system before.

**Scheme 2:**
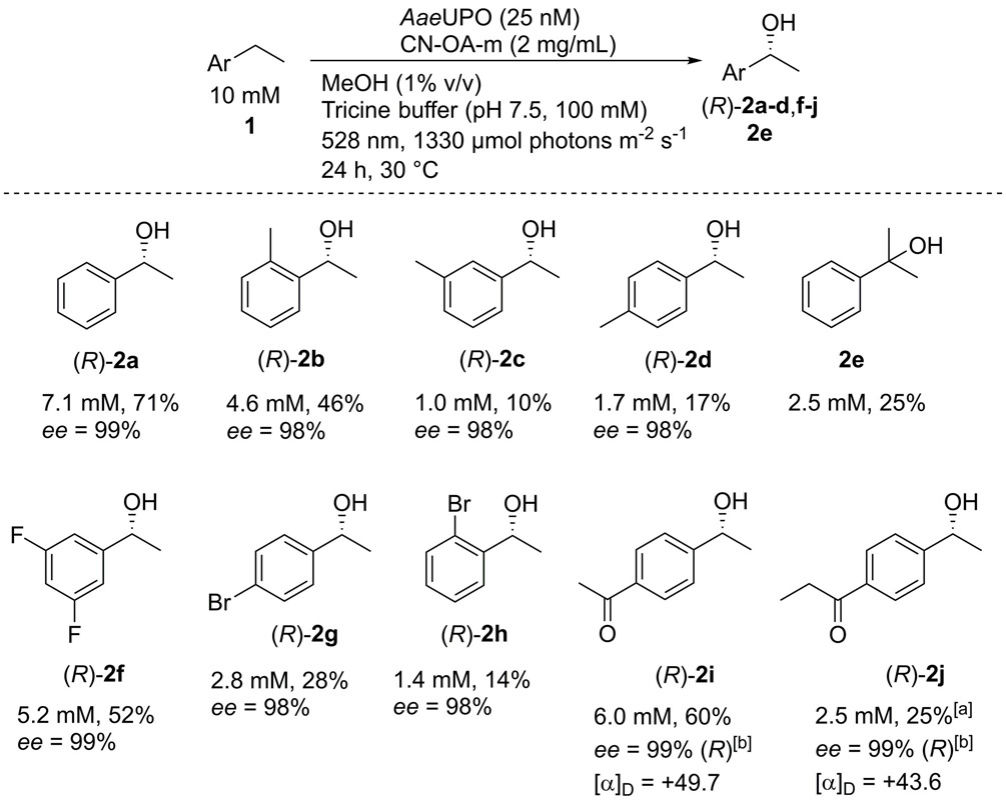
Substrate scope of *Aae*UPO using H_2_O_2_ generated by CN‐OA‐m under green light irradiation. Absolute configurations were determined by reference material except otherwise stated. [a] Based on external calibration curves of **2 i**. [b] (*R*)‐Enantiomer determined by measurement of the specific rotation (20 °C, *c*=1.00, CHCl_3_) and comparison to literature.

Ethylbenzenes bearing a methyl substituent in the *ortho*‐ or *meta*‐position were hydroxylated with 99 % regioselectivity at the ethyl group to give the desired chiral alcohols (*R*)‐**2 b**,**c**. This ability to distinguish between a methyl and an ethyl group has not been reported before. A possible explanation for this selectivity might be a preferred formation of the secondary intermediate radical over the primary radical. Acetophenone substituted with ethyl in the *para*‐position (**1 i**) allowed one to access a bi‐functionalised chiral hydroxyketone **2 i**, which is otherwise difficult to make. The same is true for **2 j**.

Recycling experiments further showed that CN‐OA‐m can be reused by centrifugation and one washing step with water. CN‐OA‐m was reused three times after drying at room temperature (Figure S49–S51). Transferring the photo‐chemo‐enzymatic hydroxylation from a total volume of 1 mL in 1.5 mL glass vials successfully to a larger scale (7 mL volume, 10 mL tubes) in another photoreactor (provided by GlaxoSmithKline, S5),^[16]^ showed the robustness and reproducibility of the approach. The hydroxylation of **1 a** worked equally well giving up to 7.5 mM of (*R*)‐**2 a**.

Recently, photo‐chemo‐biocatalytic cascades were reported combining a photoredox oxidation of ethylbenzene with an enzymatic reduction.^[17]^ In a related approach a photo‐chemo‐biocatalytic cascade that yields the corresponding (*S*)‐enantiomers was set up by taking advantage of the chromoselective activation of CN‐OA‐m (Scheme 3). The blue‐light mediated oxidation of **1 a** to **3 a** proceeded smoothly in KPi buffer. The resulting ketone (**3 a**) was stereoselectively reduced using an alcohol dehydrogenase (ADH‐A) from *Rhodococcus ruber* in the presence of NAD^+^ as cofactor.^[18]^ The optimized two‐step one‐pot procedure led to 2.5 mM (*S*)‐**2 a** with an *ee* of 93 %. The lower *ee* obtained in the photochemo‐enzymatic cascade compared to previous reports by ADH‐A (*ee* 99 %),^[19]^ can be explained by the formation of a small amount of *rac*‐1‐phenylethanol during the photocatalytic reaction under blue light irradiation (Table S3). This cascade represents a stereocomplementary pathway compared to the pathway with *Aae*UPO using the same photocatalyst. Interestingly, it was noticed that MeOH was not required for the reaction to hydroxylate ethylbenzene with *Aae*UPO. Without MeOH the same concentration of product was detected. Thus, the reaction is possible without a sacrificial electron donor like MeOH or formate, which is in contrast to some examples reported in literature.[^14a^, ^20^] For practical reasons, MeOH was still used since it simplified the preparation of stock solutions of the hydrophobic substrates. To test whether the cascade can also be transferred to other substrates, *para*‐ and *ortho*‐bromo‐substituted ethylbenzene (**1 g**, **1 h**) were investigated: Using the blue‐light pathway, (*S*)‐**2 g** was obtained with an *ee* of >99 % (1 mM) and (*S*)‐**2 h** with an *ee* of 94 % (1.4 mM) (Figure S32 and S37).

**Scheme 3:**
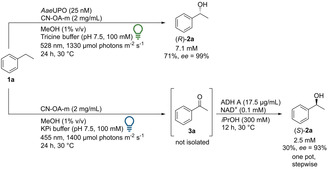
Light‐driven enantioselective oxyfunctionalizations of **1 a** by using chromoselective CN‐OA‐m and *Aae*UPO or ADH‐A.

To the best of our knowledge this is the first example in which it was possible to utilize the same photocatalyst to either oxidize an organic substrate or to provide in situ formed H_2_O_2_ without photocatalytic oxidation of the substrate, all controlled only by the choice of the wavelength.

In summary, we showed that electron holes with different oxidation potentials can be generated using a carbon nitride material by simply changing the photon energy. In the presence of blue light this enables the oxidation of ethylbenzene to acetophenone in an aqueous solution. Using green light, the organic substrate does not react and only H_2_O_2_ is formed. This was the key for designing chromoselective photo‐chemo‐enzymatic cascade reactions. Selective hydrogen peroxide generation enabled the hydroxylation of ethylbenzene to give (*R*)‐1‐phenylethanol (*R*)‐**2 a** using an UPO, whereas the photocatalytic oxidation to acetophenone was coupled with an enantioselective reduction to (*S*)‐1‐phenylethanol (*S*)‐**2 a** by an ADH. Additionally, low‐energy photons (green light) increased the stability of UPO compared to blue light, which permitted the expansion of the substrate scope of this enzyme. Controlling the outcome of a photocatalytic reaction merely through the choice of wavelength employed presents exciting new options in reaction design and could be an important new tool for controlling reactivity and stereoselection in organic synthesis.

## Conflict of interest

The authors declare no conflict of interest.

## Supporting information

As a service to our authors and readers, this journal provides supporting information supplied by the authors. Such materials are peer reviewed and may be re‐organized for online delivery, but are not copy‐edited or typeset. Technical support issues arising from supporting information (other than missing files) should be addressed to the authors.

Supplementary
